# ‘The wrong’ kind of students or ‘Santa’s workshop’? Teaching practices for newly arrived migrant students in Swedish upper secondary VET

**DOI:** 10.1007/s12186-023-09313-2

**Published:** 2023-03-09

**Authors:** Enni Paul

**Affiliations:** Dept. of Special Education, Stockholm University, Albanovägen 28, 114 19, Stockholm, Sweden

**Keywords:** Teaching practices, Newly arrived migrant students, School-based vocational education, Second language learning, Language introduction programme, Practice architectures

## Abstract

At some upper secondary schools in Sweden, newly arrived migrant youths can attend vocational courses while studying in the language introduction programme. The teaching practices in relation to language learning for newly arrived migrant students in this kind of school-based VET and how these practices are conditioned are investigated in the article. Eight VET-teachers were interviewed, and the narratives were analyzed using concepts from the theory of practice architectures. Three teaching practices in relation to language learning were identified within the broader project of teaching newly arrived students in VET: i) Swedish language first, ii) second language learning-in-action, and iii) joint VET and second language teaching. These practices were in turn connected to three different approaches to language learning in VET: language learning understood as a) segregated skills instruction, b) as happening ‘naturally’ while participating in VET-practice, c) integrated in VET but requiring explicit instruction and daily interaction with Swedish-speaking students. A conclusion drawn from the study is that newly arrived migrant students are provided unequal opportunities for development of vocational knowing and language competences in Swedish upper secondary schools depending on local conditions. The results also show how economic resources and support from school-leaders provides conditions for re-shaping teaching practices.

## Introduction

A political response to migration has been the promotion of VET-education as an integration measure (Calmfors & Gassen, 2019; Jeon, 2019), revealing underlying assumptions of VET as an easier educational pathway, tied to a hierarchical view of knowledge (Berglund & Henning Loeb, 2013; Henning Loeb, 2019). Such (mis-)conceptions obscure that just like knowing in other areas, VET contains specialized language and theoretical dimensions – albeit often embedded in practical tasks, as *knowing how* rather than *knowing that* (Carlgren, 2015; Ryle, 1963). Arguably, vocational knowing is not more accessible for newly arrived migrants than content matter in other areas/subjects (Henning Loeb, 2019; Salerno & Kibler, 2015).

The conception of VET as an especially suitable social inclusion route for migrants and marginalized groups through a ‘learning to labor’-measure, are enmeshed in neo-liberal discourses where the triumvirate of establishment, employability and (national/majority) language skills are framed as an individual responsibility (cf. Fejes, 2010; Fejes & Dahlstedt, 2017; Flubacher &Yeung, 2016). In this way, the idea of establishment in society through language learning for work, sidetracks attention from discrimination and hindering structures (Avis et al., 2022), as monolingual ideologies operate in re-producing a deficit-view where the migrant’s language use is construed as a problem (Ruiz, 1984), concealing heterogeneity in society (Blackledge, 2000).^1^

Sweden, like many other countries in Europe, has in recent years seen a course-change in migration discourses, with calls for language testing, tightening of migration laws and the growth of xenophobic political parties (Dahlstedt & Neergaard, 2019; Yantseva, 2020). This shift became palpable in the wake of the 2015 European refugee crisis, which in the Swedish case also put upper secondary schools in the spotlight as the language introduction programme (LIP) – targeting newly arrived migrants aged 16–19 years – became the fourth largest, encompassing 10% of the students enrolled in upper secondary school in 2016–2017 (SNAE, 2018a).^2^

At some upper secondary schools’, vocational courses have been offered to newly arrived migrant students, which means that VET-teachers encounter students they have little previous experience of teaching (Ashgari & Abraham, 2022). These teaching practices are underexplored. On the whole, migration in connection to VET is still emerging as a research field (e.g. Moreno Herrera et al., 2022). This article therefore seeks to contribute to this growing field by investigating VET-teachers’ narratives of teaching practices for newly arrived migrant students in VET in relation to language learning, through exploration of the following research questions: *How is language learning construed within the teaching practices? What conditions shape these practices?*

## Newly arrived migrant students in the Swedish upper secondary school

Newly arrived migrant students (NAMS) between the ages of 16 and 19 years are placed in the language introduction programme (LIP) if they have been residing less than four years in Sweden; do not fulfill the requirements for a national upper secondary programme; and the requirements of Swedish language skills. The objective is to rapidly learn Swedish and qualify for an upper secondary programme (SNAE, 2016). However, approximately only a third of the students transition to a national upper secondary programme within four years (SNAE, 2018a). Some of the students are in a precarious situation; since asylum seeking youth (who have reached 18 years of age) have to get an employment within six months of finishing upper secondary education in order to be eligible for a residence permit (SFS 2016:752; SFS 2018:756). This means that there is a time limit for learning the language and earning the qualifications necessary for a national upper secondary programme, and for some of them also for securing employment.

Each local school has the right to decide what courses to offer and how to organize LIP, with Swedish as Second Language (L2) as the only mandatory subject. At some schools, VET-courses are offered to these students. It is the narratives of VET-teachers who teach these kinds of courses that are investigated in the article.

## Previous studies on newly arrived migrants, language learning and VET

Students with migration background face many obstacles in VET (e.g. Avis et al., 2022; Chadderton & Edmonds, 2015; Jørgensen et. al., 2021; Rosvall et al., 2018). Some of these hindrances concern insufficient opportunities for language learning (Choy & Wärvik, 2019; Teräs, 2013), for instance due to lack of access to communicative situations during workplace-based learning (Sandwall, 2010).

VET-teachers, just like teachers in other areas/subjects, use specialist language and discourses from their vocations in interaction with their students (Green et al., 2017). In order to convey conceptual, procedural and dispositional knowledge that is abstract, tacit or learnt by experiencing in VET, analogies are commonly used (Fillietaz et. al., 2010), which points to language being used in particular ways in VET.

However, VET-teachers seldom describe language and literacy instruction as part of their teaching tasks (Hellne-Halvorsen, 2014; Ivanic et al., 2009; Paul, 2021). This has to do with language and literacies in many vocations, and consequently also in VET, being ‘invisible’ (Karlsson, 2006). However, with students with migration backgrounds participating in VET, language as a feature of vocational knowing moves to the forefront as the students have to learn the new language and VET-content through the medium of the new language, which is placing high demands on VET-teachers (Henning-Loeb et al., 2018; Teräs, 2013). In this sense, it could be claimed that all VET-teachers are language teachers, but for this to be realized VET-teachers need linguistic competences and pedagogical strategies for guiding students’ language and literacy development (Blixen & Hellne-Halvorsen, 2022). Seminal pedagogical-didactical work points to language-oriented content teaching (e.g. Hajer, 2018); contextualizing and scaffolding in communicative situations (e.g. Gibbons, 2002); and an explicit focus on both everyday language and specialized language connected to content-matter (e.g. Lindberg, 2007) as successful routes to L2-learning – however, VET has seldom been regarded in this kind of research.

However, in several studies, Henning-Loeb (2019, 2020) and Henning-Loeb with colleagues (2016; 2018), show how shortcomings in educational organization, teaching methods and cooperation between teachers hinder the development of vocational knowing and language development for students with migration background studying in vocational introduction programmes or complementary adult VET. How teachers and school leaders view L2-speakers’ potential for developing vocational knowing and language competences affects the teaching provided and thus the opportunities for learning. Also, major differences in allocation of economic resources leads to a lack of equivalence in the students’ opportunities to develop language competences and vocational knowing (Henning Loeb, 2019, 2020).

Despite a multilingual turn in research (May, 2014) – emphasizing that migrant students’ different languages and multilingual competences can be used as resources for meaning-making and communication (e.g. García & Wei, 2014) – these ideas seem to not have altered school praxis. Instead, research makes it clear that NAMS’ previous knowledge, languages and experiences are rarely utilized in LIP and that the students are viewed according to the lines of a deficit-discourse, offered little to no contact with majority-language speaking students and treated like a homogenous group despite heterogenous backgrounds and needs (e.g. Brännström et al., 2019; Bunar & Juvonen, 2021; Hagström, 2018; Sharif, 2017).

In summary, prior research about LIP and its equivalents internationally do not focus on VET-courses, and the existing studies of VET for students with migrant backgrounds concern adults (e.g. Choy & Wärvik, 2019; Sandwall, 2010; Teräs, 2013); students who have moved on from LIP to the next step in the education system (e.g. Henning-Loeb et al., 2016); or general subject teachers’ experiences (Ashgari & Abraham, 2022). When pedagogical practices in ‘regular’ upper secondary VET have not been transformed to address the experiences of an increasingly heterogenous student population (Rosvall et al., 2018), while VET at the same time is promoted as a social inclusion measure (Calmfors & Gassen, 2019; Jeon, 2019), makes VET-courses offered to newly arrived students in upper secondary school an interesting case.

## Theory of practice architectures as a conceptual framework

This article takes an interest in the site-based teaching practices in the language introduction programme within the broader project of teaching newly arrived migrant students in VET by drawing on the theory of practice architectures (Kemmis & Grootenboer, 2008; Kemmis et al., 2014). What people – in this case VET-teachers –say/think, do and how they relate to others are situated within practice(-s), directed towards specific purposes.

The bundles of ‘sayings’, ‘doings’ and ‘relatings’ take place in intersubjective spaces where individuals meet one another: as interlocutors in semantic space; as embodied beings engaged in interaction in physical space; and as social beings in interrelationships in social space (Edwards-Groves & Grootenboer, 2017; Kemmis et al., 2020). Practices are site-based, meaning that they both shape and are shaped by particular cultural-discursive, material-economic and social-political arrangements, conditioning what is perceived as knowledgeable and meaningful action at a specific site (Kemmis et al., 2014; Mahon et al, 2017). For a practice to be changed, the arrangements holding the practice in place also have to be transformed.

In the semantic space encounters are formed through the medium of language and specific discourses, that are prefigured by cultural-discursive arrangements (Kemmis et al., 2014). In other words, what is said by the VET-teachers is influenced by for instance dominant discourses in the national curriculum; vocational traditions and pedagogical discourses (Green et al., 2017). Encounters also take place in physical space–time, realized in the medium of activity, work and use of tools and objects – expressed as doings – conditioning and conditioned by material-economic arrangements (Kemmis et al., 2014). For instance, what learning activities VET-teachers design and what teaching materials they use are enabled and constrained by the resources allocated for teaching at a particular school (such as schedules, the physical set up, access to teaching material and equipment). In the social space people encounter one another through the medium of solidarity, power and belonging, which are prefigured by social-political arrangements forming rules, norms and roles shaping how people relate to one another (Kemmis et al., 2014). This encompasses for instance how the VET-teachers relate to and understand their students, how they cooperate with colleagues and hierarchies at a school.

## Method and materials

The empirical material is part of a planned practice-based research project; however due to the COVID-19 pandemic the on-site parts of the study could not be realized. Still, interviews with VET-teachers and Swedish as a Second Language (L2)-teachers were carried out. For this article the interviews with VET-teachers have been analyzed as there is a lack of research on how VET-teachers handle the new situation of teaching newly arrived migrant students.

Schools that according to their homepages had both the language introduction programme and national VET-programmes/vocational introduction programmes, and thus meet the conditions of being able to offer vocational courses for the NAMS, were contacted in four different municipalities in different parts of Sweden. As it turned out, the majority of the schools did not offer VET-courses for the students in LIP or had stopped offering VET-courses to the students. All schools where the school leader responded that they had this combination also had VET-teachers willing to participate in the study.

The data for the study consists of transcripts from eight audio-recorded semi-structured interviews (Kvale, 1997) with VET-teachers teaching newly arrived students enrolled in LIP. The interviews averaged between 40 min to an hour, and were conducted through an online platform or by telephone. The interviews began with questions about the VET-teachers vocational and teaching background; the general organization at the school; and cooperation with other teachers (especially L2-teachers and mother tongue instructors), before turning to center upon their teaching of newly arrived students in VET and especially how they worked with language and literacies in VET in general and for these students in particular. The VET-teachers were also asked to highlight what they found to work well in their teaching and what obstacles they faced.

Two of the interviews were conducted in pairs, since these VET-teachers worked at the same school in co-operation with each other; the other four teachers were interviewed individually. All in all, the teachers represented six different schools in three different municipalities. At all the schools, except one, courses in just one type of VET-programme were offered to the NAMS. The interviewed teachers represent the Building and Construction Programme (*N* = 4), Health and Social Care Programme (*N* = 2), Restaurant Management and Food Programme (*N* = 1), and the Handicraft Programme (*N* = 1). Quotes from the transcripts have been translated to convey meaning as precisely as possible to the teachers’ (Swedish) utterances.

Prior to the interview all teachers were informed about the study, ensuring that they consented to participation. All the names in the article are pseudonyms and information that could reveal the municipality or school in question has been left out. The Swedish Ethical Review Authority has granted approval of the study (ref. no. 2020–06,297).

### Analysis

The interviews provided narratives that subsequently have been analyzed as ‘sayings’, ‘doings’ and ‘relatings’ of the teaching practices.^3^ When analyzing the transcripts, what was identified as sayings, doings, relatings, as well as arrangements conditioning these, were separated and marked. Analytical tables were formed for each school (see Table 1 for an example) during this phase.

**Table 1:** Example of an analytical table; School 4 (simplified for the article)

Selection of quotes from the interviews	Arrangements
Sayings: “We thought they will learn the language while studying and at the workplaces.” “There’s so much theory in Health Care.” “You have to be able to communicate with patients and with your colleagues. It’s important for patient safety.” “The curriculum goals just weren’t reachable in these circumstances as it turned out.”	Cultural-Discursive: national curriculum with VET-courses and examination goals, vocational standards and regulations, migration law, discourses about language learning, discourses about the vocation and VET
Doings (emerging in the interviews): “We worked a lot in the method room.” “We did a lot of role-playing so they could practice communication.” “We made compendiums so it would be easier. Like, shortened versions of the content in the textbook.” “We found workplaces for all of them [for workplace-based learning].”	Material-Economic: buildings and classrooms, time allocated for courses, space for collaboration with L2-teachers, (lack of) teaching materials available for newly arrived students in VET-subjects, workplaces prepared to take NAMS
Relatings: “They are wonderful students.” “Many say they want to become nurses in the future, so they really want to do this.” “We [L2-teacher and VET-teachers] had some content-integrated themes that we planned together” “They [the students] were appreciated at the workplaces and the elderly really liked them.”	Social-Political: cooperation with L2-teachers, students understood as motivated, engaged and suitable for the vocation, Power relations in relation to the time-allocated and the time-framework of migration law

This was followed by a sorting of similarities and differences between the six schools informed by the analytical question of: *What approach to language learning is discernible at this school? What kind of local teaching practice is this an example of within the larger goal of teaching newly arrived students in VET?* Eventually, through a thematic analysis (Braun & Clarke, 2006), and re-working the analytical tables (see Table 2), three overarching themes representing three teaching practices interwoven with a particular approach to language learning were formed, where the utterances from teachers representing four of the six schools fell under the same heading and the other two formed their own local practice respectively.

**Table 2:** Analytical table in the last step (simplified for the article)

Quotes (in selection)	Arrangements (in selection)	Approach to language learning	Teaching practice
*Theme 1*			
S: “I am not a language teacher” “They have to learn the language”	C-D: curriculum, construction standards, deficit-discourse	Segregated skills instruction	Swedish language first
D: “I made a work sheet.” “Then we had a test”	M-E: two different campus-sites, lack of teaching material for NAMS in VET		
R: “They aren’t interested in learning” “The politicians think everyone can just go out and hammer and saw”	S-P: no cooperation between VET and L2-teachers, NAMS seen as unmotivated, solidarity with VET-community		
*Theme 2*			
S: “I’m not an expert on language.” “We thought they will learn the language while studying and at the workplaces.”	C-D: curriculum, vocational norms, idea of vocational language developing ‘by itself’ in VET	In ‘natural’ communication while participating in VET	Second language learning-in-action in VET
D: “We don’t have the time to stop” “Now I put words to everything I do.”	M-E: allocated teaching time, space for meetings with L2-teachers		
R: “They are wonderful students.” “It’s unfair, because they work so hard.”	S-P: Advice from L2-teachers, power relations in relation to the time-allocated and the time-framework of migration law		
*Theme 3*			
S: “Because I’m not a language teacher” “Everything is about the language and the vocation at the same time.”	C-D: Curriculum, quality norms in VET, resource-perspective	Content integrated language instruction in VET (explicit instruction and interaction with Swedish-speaking students)	Joint VET and language teaching
D: “We always pair them with Swedish-born students” “We invite them to come and visit.”	M-E: Economic resources for re-organization of LIP, dual teacher system		
R: “What was important to me […] was to protect VET and not to compromise with the quality” “I bring my vocational competence and she has her expertise”	S-P: Solidarity with VET-community, VET and L2-teacher cooperate in teaching		

## Results

The results are presented under three headings, each representing the teaching practice that emerged in the analysis of the teachers’ narratives with specific ways of construing language learning within each of them: i) Swedish language first, ii) Second language learning-in-action, iii) Joint VET and language teaching. The arrangements holding these practices in place, conditioning them, are presented in each section and then developed further in the discussion. It was found that neither cooperation with mother tongue instructors or the offer of multilingual classroom assistance in relation to VET existed at any of the schools, despite policy documents (SFS 2010:2039, 9 ch. 9 §) and research highlighting promotion of language learning by using multilingual students’ languages as resources for learning (García & Wei, 2014).

### Swedish language first, vocational practice after

‘Sayings’ by almost all VET-teachers included statements of not being ‘a language-teacher’. At one of the schools, the two VET-teachers interviewed, both working in the Building and Construction Programme, made this comment in connection to expressions that showed a frustration with being assigned the task of teaching newly arrived migrant students.

When we got this task of having language introduction students, at least on my part, I thought, I’m not a language teacher. I’m not a Swedish [language] teacher. I’m a VET-teacher. But it didn’t matter what we said. We just had to do it and make the best of it.

Simon (Building and Construction Programme)

The VET-teachers had protested against the decision of teaching the students, but had not been heard. In the interview they expressed annoyance directed at the school-leaders and the politicians in the municipality. The teachers’ reluctance towards teaching these students can be explained when considering the material-economic and social-political arrangements conditioning their teaching. At this particular school, VET and general subjects (including L2) were placed at two different geographical locations – a ‘VET-campus’ and an ‘academic campus’ – which the students travelled between. This spatial division created hindrances for collaboration between VET- and L2-teachers. There was lack of organisational support from the school leaders, but also a lack of clarity in what the teaching assignment entailed. Therefore, the VET-teachers had to figure out what teaching this student group meant on their own.

Sure, many of them [NAMS] might be really good at the practical parts, but that’s not what we are doing. They are language introduction [students] and they have to learn the language.

Jacob, (Building and Construction Programme)

This view of the NAMS as language learners first and foremost, and thus that the task should be about teaching language before “practical parts”, had consequences for the ‘doings’, namely what kind of teaching activities were chosen, as well as for how the teachers ‘related’ to the students. Unsupported by the school leaders and with no space provided for working together with L2-teachers, one of the VET-teachers explained how he had gotten the idea for teaching these students from his wife, who was a primary-school teacher.

I sort of copied her and things she does with her first, second and third graders. I made a work sheet with pictures of different tools and what the tool is called. And then they should fill out their own worksheets, and on another one draw lines between the picture and the word. A bit childish stuff like that. And then they had to study these worksheets and learn the name of the tools. […] Then we had a test. […] The idea was that they could do something in the workshop after they passed the test. But that was stopped by… To put it simply, the tests were a catastrophe. So, it didn’t feel meaningful. They aren’t interested in learning.

Simon, (Building and Construction Programme)

Swedish skills – in the form of being able to name tools in a test – functioned as a checkpoint hindering access to the VET-workshop and the later possibility of entering workplace-based learning. The teachers stressed the importance of understanding written security instructions in the workshop as a reason for having to have a certain command of the language (cf. Henning-Loeb, 2019). But these expressions about ‘doings’ – filling out worksheets with pictures of tools, memorizing the names and then writing a test – did not prove to be successful and the students’ reaction to the teaching activities, as narrated by the teachers, had consequences for how the teachers came to understand and relate to them, with the VET-teachers revolving around how the failure on the tests showed that the NAMS were unmotivated and uninterested in learning, hence being the ‘wrong kind’ of students for VET.

Behind this teaching activity traces of cultural-discursive arrangements can be found in the form of ideas about L2 as acquired through training of particular and delimited language skills – words for tools in this case. Also, a deficit-discourse (Ruiz, 1984) where students’ difficulties are understood as depending on the individual’s (lacking) motivation, migration-background and language skills in line with monolingual norms, came forth in the ‘sayings’. This shows how cultural-discursive arrangements expressed in the semantic dimension, conflate with material-economic and social-political arrangements, conditioning what is manifested in both the physical dimension and the social dimension – in what type of teaching material was created and how the failure on the tests had repercussions: i.e. not getting access to the VET-workshop and later workplace-based learning, and also influencing the interpersonal relations between the VET-teachers and the students.

Nevertheless, despite their expressions of negative feelings towards teaching NAMS, the VET-teachers were concerned for their future. Since the students were not passing the VET-courses and thus not getting access to workplaces during the workplace-based learning, it could diminish their chances of getting employment and therefore also securing a residence permit.

Simon: It's really hard for us too, because if they don’t get employment then they are kicked out of the country. And that’s crazy as well. Totally wrong.

Jacob: Why not assign them to an academic programme instead, huh. The politicians think that everyone can just go out and hammer and saw. But there is a construction standard. There are regulations. We can’t ignore them.

Simon: Right. I feel pride in my vocation.

(Building and Construction Programme)

This quote and similar expressions in the interview point to how national cultural-discursive arrangements in the form of the migration law, the curriculum and vocational discourses – as well as local cultural-discursive arrangements for instance voiced through the medium of the ‘VET-language test’ inspired by ideas of L2-learning within a skills-based approach – constrained the future possibilities of the students, when interwoven with material-economic and social-political arrangements. The political discourse in Sweden where VET is portrayed as an easier learning path (Berglund & Henning-Loeb, 2013) was fiercely contested by the teachers, by holding high standards and regulations in the vocational construction field. In this sense, the teachers expressed strong solidarity with the community of vocational practitioners, or as they put it: “I feel pride in my vocation”. Nonetheless, following the assessment criteria in the curriculum and upholding vocational standards meant facing an ethical dilemma, since failing the students in the VET-courses and not letting them enter workplace-based learning could have major negative consequences for the NAMS’ future, which the teachers had to handle in their daily interaction with the students.

#### Second language learning-in-action

The second teaching practice – *second language learning-in-action* – was manifested in the narratives of teachers representing four different schools. In this teaching practice VET-teachers worked on the same campus as L2-teachers (even if they sometimes had their workspaces in different parts of the campus) and mostly belonged to the same teacher team. Sometimes the teachers also planned and cooperated on common content-integrated themes, with lessons on a particular topic held in both VET and L2-classes. Furthermore, the VET-teachers described that they could get advice from the L2-teachers.

She [the L2-teacher] has given tips about how I can work with language. So, now I really make sure to put words to everything I do. Like, I’m really using the right word instead of just pointing at things saying ‘take that’.

Samuel (Handicraft Programme)

This work relationship with L2-teachers had provided a new way of thinking about and working with language for the VET-teacher, thus displaying how local material-economic arrangements providing time and space for interaction between different teacher categories support social-political arrangements in enabling collaborative exchange, with the VET-teacher becoming more conscious about language use. Nonetheless, the VET-teachers explained that they taught the students similarly to Swedish-born students in the national VET-programme or vocational introduction programme.

I teach them just like I teach my other students. Sure, I have to explain more and it takes more time but there isn’t that big of a difference in the way I teach. It’s still the same stuff they have to learn.

Andreas (Building and Construction Programme)

VET-content was placed in the foreground as the “stuff they have to learn”, regardless of being NAMS or ‘regular’ students, displaying how the teachers’ came to recognize the newly arrived migrants as similar to the Swedish-born students in terms of what they needed to learn, and thus being provided the same kind of teaching activities. The main difference was the teachers emphasizing that they needed to ‘do’ more explicit language work, for instance when giving instructions or creating additional teaching material, and that the students needed more time. However, the approach to language was largely based on the idea of Swedish as well as vocational language developing while participating in VET-lessons, tying in with cultural-discursive arrangements in the form of viewing language learning as occurring ‘naturally’ while participating in communicative situations.

The VET-teachers explained that it was not the vocational specific words that created difficulties for the NAMS. Since these were also new to Swedish-born students, the VET-teachers were used to emphasizing VET-specific language in their teaching and creating activities where the students were afforded chances to learn to use the vocational concepts. Rather, it was the general language that caused difficulties for the NAMS according to the teachers (cf. Henning-Loeb et al., 2016; Lindberg, 2007). Some of the VET-teachers explained that their usual strategies of using metaphors (cf. Fillietaz et. al., 2010) did not work with these students as they often did not understand the cultural context of the metaphors, and that the general language became more difficult and abstract in the advanced VET-courses.

The problems begin when they come to the more advanced courses. […] You explain more advanced stuff and then you use words that aren’t the most common ones to explain. And we don’t have the time to stop and take it really slow with them because the course time runs out, so this is a problem.

John (Restaurant Management and Food Programme)

It was in relation to expressions like this that some of the VET-teachers said that they were not language teachers, stating that they did not know how to or view it as their task to develop the students’ general (Swedish) language competences. This illuminates that the cooperation with L2-teachers mostly stayed at the level of receiving (some) advice, but did not entail a deeper collaboration with more profound changes in the teaching practices.

The VET-teachers furthermore felt that they weren’t able to “take it really slow” in order to make sure the NAMS understood the vocational content and developed the required vocational knowing, leading to the students seldom passing advanced level courses. This time-constraint was conditioned by cultural-discursive arrangements in how VET-content is divided into courses in the national curriculum (Gy, 2011) as well as teaching traditions formed in VET, interwoven with local material-economic arrangements shaping how the courses were organized at the school-sites and how teaching time was allocated for the courses.

Mary: Now in hindsight, I think we were naïve. We thought they would learn the language while studying [VET-courses] and at the workplaces. And sure, they did. But not enough to be able to pass the courses.

Sara: They are wonderful students, very engaged and they want to work in health care. But there just wasn’t enough time to get them were they need to be.

(Health and Social Care Programme)

The dominant discourse of workplace-based learning as a successful path for language learning and establishment in society for migrants, had influenced the two Health and Social Care-teachers (cf. Sandwall, 2010). But as the quote shows, they had revised their initial beliefs as it was not leading to the outcomes they had anticipated. Sara and Mary, like the other VET-teachers within this practice, portrayed the students as hard-working and motivated. But the narrow time-frame in the VET-courses formed a major hindrance for the students’ educational trajectory.

Within this practice the lack of time was connected to abstract and theoretical aspects of the vocational content and communicative situations in the vocational practice – all requiring a certain language level “to be able to pass the courses”. The teachers had come to view this language level difficult to acquire during the VET-courses and during workplace-based learning within the time-frame allocated at the local schools for each VET-course. This shows how social-political arrangements were bundled up with material-economic arrangements as the identified needs of these students had to stand back for the conditions in the physical space–time like the school’s organization of the courses and the teachers’ scheduled teaching hours. But it also shows, how the ideas of language learning happening while participating in VET meant that the teaching practices in terms of language instruction in VET had not been accommodated to this student group’s needs of support in developing ‘general abstract language’ (Lindberg, 2007) needed for passing the advanced VET-courses (Henning-Loeb et al., 2016).

Lack of time was also put in relation to the migration law, with the teachers expressing concern for the consequences the students might face following their decisions of failing them in VET-courses. But as professional judgement formed in relation to the criteria in the national curriculum, vocational standards and norms as well as solidarity with the vocational and teacher communities shaped what actions the teachers interpreted as possible. This arrangement pressed them to make painful decisions and they were well aware of the grave outcome the decisions might have for some of the students’ futures.

#### Joint language and VET teaching

The last practice identified – *joint language and VET teaching* – was discerned in the narrative of one teacher representing one school. This practice revolved around a dual-teacher system with a VET and a L2-teacher teaching a mixed student-group consisting of NAMS in the language introduction programme and Swedish-born students in the vocational introduction programme.

Before placement, the students were interviewed in order to determine if they might be interested in a vocation, and then invited to visit VET for a day or two, before given the option to enroll in a regular LIP or study vocational courses while in LIP.

The aim is that these students become a part of the vocational introduction group during their first language introduction year to shorten the time and increase the motivation.

Thomas (Building and Construction Programme)

Such expressions of ‘doings’ in the organizing of the teaching groups emanated from the idea of “a fourth year first” in order to combat getting stuck in the language introduction programme. Also, the quote highlights that the students’ motivation was put in relation to getting the opportunity of being included in a group with Swedish-born students.

During the first year the NAMS studied the same VET-courses as the students in the vocational introduction programme; the rest of the time they studied Swedish as a Second Language. After the first year, the students were transferred to a national VET-programme as long as they had acquired compulsory school 7^th^ or 8^th^ grade level Swedish skills or to the vocational introduction programme. After transitioning they would follow that programme’s curriculum, but with additional L2-instruction twice a week. In this sense the school used the flexibility in the curriculum to form a particular pathway through the education for the NAMS.

The VET-teacher explained that he at first had hesitated about the idea of teaching these students in VET, fearing VET would be made into a “light version”.

What was important to me, when we started with these students, was to protect VET and not to compromise with the quality. Because that is what we in VET fear when there are new target groups. So, I was very fastidious in that if we work together, the quality has to be as high [as in regular VET].

Thomas (Building and Construction Programme)

The ‘relatings’ discernible in the quote point to social-political arrangements displaying both solidarity towards the VET-community as well as illuminating power relations in schools exhibited in the fear of the quality of VET being lowered through decisions made by teachers/school-personnel not part of a VET-community. But when talking to the L2-teachers the VET-teacher had come to understand how this way of teaching could benefit both NAMS and Swedish-born students, and a teaching practice was formed starting from the vocational content as the departure point. The L2-teacher would join the VET-teacher in order to support language learning during VET-classes and then pick up on what was done in VET to use it as a theme in the L2-classroom. This way of forming teaching can be seen emanating from cultural-discursive arrangements in the form of theories of content-based language instruction and other similar theories of L2-learning where language-oriented learning activities are provided from within the subject matter at hand (e.g. Gibbons, 2002; Hajer, 2018).

As stated earlier, almost all VET-teachers said that they were not language teachers. Here this ‘saying’ was an expression of how the cooperation with the L2-teacher had provided the VET-teacher with new ideas for teaching.

It’s fun when you get all these ideas to use on how to get through with the language, like different methods and ways of working. Because I’m not a language teacher. But I bring my vocational competence and she [the L2-teacher] has her expertise.

Thomas (Building and Construction Programme)

The close cooperation between VET and L2-teachers was supported by the school leaders and also made possible by the politicians in the municipality allocating money for the introduction programmes according to the VET-teacher. This shows how material-economic conditions at the municipal level can support the reshaping of teaching practices at local schools by providing conditions for making it possible to rethink the organization at the school, which in turn made room for new ways of ‘doing’ teaching. The allocation of teaching time for L2-teachers to work in the classroom/workshops together with VET-teachers, and also allocating more teaching time for the VET-teacher to work with the mixed group, thus supported social-political arrangements where the VET-teacher and L2-teacher could learn from and complement each other in teaching the NAMS by combining their respective expertise areas, and also provided new ways of relating to the students. This school was the only site where NAMS were placed in the same class as Swedish-born students, and where the teaching was organized as to afford scaffolding between peers.

We always pair them with Swedish-born students. […] It’s difficult also for the Swedish-born students to explain what something means. It’s not easy to explain, but that is part of Swedish language-course goals to communicate, to teach someone something you know. So, the Swedish language teacher gets happy about it. And I get super happy because it’s like Santa’s workshop. There is activity everywhere and everything is about the language and the vocation at the same time*.*

Thomas (Building and Construction Programme)

The saying: “everything is about the language and the vocation at the same time” points to VET and language development being regarded as mutually important within this teaching practice (cf. Henning-Loeb et al., 2016; Hajer, 2018). Additionally, according to the VET-teacher the Swedish-born students benefited from studying with NAMS, which was made sense of in relation to the national curriculum’s course goals in the subject of Swedish (as a first language), pointing to both cultural-discursive and social-political arrangements, with ideas connecting to a ‘language as a resource’-perspective (Ruiz, 1984), where the NAMS were being viewed as assets contributing to the Swedish-born students’ learning and vice versa, even if the NAMS’ languages were not utilized in the teaching practice.

According to the VET-teacher the school had been successful in getting the NAMS to transition to a national VET-programme within a year and then receiving an upper secondary VET-diploma. Consequently, this was the only interview were time wasn’t framed as a major obstacle for the students’ learning trajectory.

## Discussion

The results show three different types of teaching practices: i) Swedish language first ii) second language learning-in-action iii) joint VET and second language teaching, each connected to an approach to language learning, i.e.: a) segregated skills instruction b) happening naturally when participating in VET or c) integrated in VET with explicit language instruction and daily interaction with Swedish-speaking students. Furthermore, the results suggest that the teaching practices might be less tied to any particular vocational area, as the Building and Construction Programme was represented in all three practices, than to local arrangements in which the practices were enmeshed (Mahon et al., 2017).

Distinctive ways of understanding NAMS in VET connected to conceptions about L2-learning were manifested. When the students are understood as the ‘wrong kind of students’ for VET (teaching practice 1), or as ‘not yet ready’ for advanced VET-courses (teaching practice 2) it largely places the blame and responsibility on the students themselves (cf. Fejes & Dahlstedt, 2017), rather than being seen as a call for adapting the teaching practices and organization of the education (e.g. Brännström, et al., 2019; Bunar & Juvonen, 2021). In this sense, the teaching practices form an obstacle for the students learning trajectory, but these structural hindrances based in monolingual ideologies are not recognized as expressions of discriminatory and exclusionary practices (cf. Avis et al., 2022).

The different ideas about language learning can be tied to how the material-economic arrangements at the local schools were intertwined with social-political and cultural-discursive arrangements, creating substantial differences in the circumstances shaping the teaching practices at the sites. For instance, at the school where there was a geographical distance between the VET-campus and the ‘academic’ campus, the spatial division hindered collaboration between VET and L2-teachers, and lead to the forming of a teaching practice influenced by an instrumental interpretation of how beginners learn a language conveying ‘infantilization’ (Franker, 2011) of the students. These school tasks seemingly affected the students’ investment in learning (Norton Peirce, 1995), which in turn reinforced the VET-teachers view of the newly arrived migrants as the wrong kind of students for VET, along the lines of a deficit-discourse (Ruiz, 1984).

In contrast, the joint teaching practice was made possible at the last school through the distribution of economic resources and support from school-leaders for new ways of organizing the education, which enhanced an understanding of the NAMS as important assets for Swedish-born students and vice versa. The teaching in this ‘Santa’s workshop’ presumably drew on discourses of L2-learning as found in work by for instance Gibbons (2002) and Hajer (2018), through scaffolding; creation of communicative situations; and explicitly addressing language learning while working on vocational content in mixed student groups. The shaping of this practice also underscores how teaching can be developed through utilizing different teacher competences, enabling changes in arrangements of practices, and illuminating the importance of support by school-leaders in providing conditions for such change (Francisco, 2017; Francisco et al., 2017). The possibilities of using the flexibility in the school policy for organizing LIP were in essence only put to use at this site.

Not accommodating the VET-courses to the NAMS so that they could both develop vocational knowing and the Swedish language and ultimately pass VET-courses, meant that the VET-teachers had to deal with an ethical dilemma as the implementation of the new restrictive migration law could lead to some of the students being deported if they did not secure employment – chances which are severely diminished by the lack of an upper secondary diploma and by having a migration background (Engdahl & Forslund, 2016). This points to how cultural-discursive and social-political arrangements in society at large affect teachers in their professional practice in how migrants are understood, treated and governed. But also, it becomes apparent that there are severe consequences in not addressing the prerequisites and needs of the students in forming the organization of the education and pedagogical-didactical tasks.

A conclusion to be drawn is that VET is not an easy path straightforwardly leading to an upper secondary diploma and thereby integration into society, as it is difficult to develop vocational knowing through the medium of a new language while also at the same time learning the new language (Henning-Loeb et al., 2018; Teräs, 2013), especially when the organization of the education and teaching is not adapted to the students’ needs (Bunar & Juvonen, 2021). Thus, as previous research makes it clear, the structural obstacles for students with migration background in VET are substantial (e.g. Avis et al., 2022; Chadderton & Edmonds, 2015; Rosvall et al., 2018). However, by making teacher cooperation possible; by allocating resources for new ways of organizing VET-courses that are rooted in pedagogical-didactical theories on L2-learning where the students’ languages and the students themselves are seen as resources rather than problems; and by arranging interaction with L1-speaking students, can provide substantial opportunities for development of both vocational knowing and language competences (e.g. García & Wei, 2014; Gibbons, 2002; Hajer, 2018; Henning Loeb et al., 2016).

Also, as argued by Blixen & Hellne-Halvorsen (2022), VET-teacher education needs to prepare VET-teachers for work with language and literacies in VET, as the VET-teachers’ sayings revealed that they did not view themselves as language teachers, even if all were in fact engaged in language teaching. This can be explained by the ‘invisibility’ of language and literacies as features of vocational knowing (Karlsson, 2006; Paul, 2021); teacher education putting little emphasis on language and literacies in VET (Blixen & Hellne-Halvorsen, 2022); and the lack of support and guidance for language teaching in VET from L2-teachers and school leaders (Henning-Loeb, 2019, cf. Paul, 2021).

Although this is a small-scale case study consisting of interviews with eight VET-teachers, thus only providing narratives of their teaching, the results could be used in a transformative sense as alternative ways of teaching that could better afford learning opportunities for the newly arrived migrant students are illuminated (cf. Francisco et al., 2017). However, to gain a fuller picture capturing the complexity of the teaching practices in VET for this group of students, further work is called for – especially research where students’ experiences and how school leaders organize for the education are explored in situ.

## Footnotes

1 In Sweden, over 30% of the inhabitants have migration backgrounds, with approx. 20% being born abroad Statistics Sweden, 2022). In upper secondary school, over 30% of the students have migration backgrounds; with Syria, Somalia, Iraq and Afghanistan topping the list of countries of birth for those born abroad (SNAE, 2018b; Statistics Sweden, 2020).

2 Since this peak, the numbers have dropped each succeeding year (SNAE, 2021).

3 Thus, it is *accounts* of doings and relatings, not documentation of actual doings and relatings unfolding in the classrooms that are analyzed, meaning that the analysis of teaching practices in this article is limited to the teachers’ narratives (e.g. Henning Loeb, 2020).

## Declarations

### Availability of data and materials

Data sharing not applicable to this article.
